# A Comparison of Plyometric and Jump Rope Training Programs for Improving Punching Performance in Junior Amateur Boxers

**DOI:** 10.3389/fbioe.2022.878527

**Published:** 2022-05-24

**Authors:** Monchai Chottidao, Chia-Hua Kuo, Shiow-Chwen Tsai, Ing-Shiou Hwang, Jiu-Jenq Lin, Yung-Shen Tsai

**Affiliations:** ^1^ College of Sports Science and Technology, Mahidol University, Nakhon Pathom, Thailand; ^2^ Graduate Institute of Sports Science, University of Taipei, Taipei, Taiwan; ^3^ Department of Physical Therapy, National Cheng Kung University, Tainan, Taiwan; ^4^ School and Graduate Institute of Physical Therapy, National Taiwan University, Taipei, Taiwan; ^5^ Graduate Institute of Sports Equipment Technology, University of Taipei, Taipei, Taiwan

**Keywords:** boxing, high school, biomechanics, motion analysis, jab-cross punch

## Abstract

Improving lower extremity sports performance may contribute to punching performance in boxers. We compared the effects of two typical boxing routines for developing lower extremity sports performance and subsequent punching performance. Twenty-four high school amateur boxers between the ages of 12 and 18 performed training at least 3 days per week. All Athletes had 3–5 years of experience in boxing training. The participants separated into two groups to receive an 8-week plyometric or jump rope training program. They performed each training program for 30 min on 3 days/week. Lower extremity sports performance in countermovement jump (leg stiffness, jump power, and rate of force development) and jab-cross punching performance (punch velocity, punch force, reaction time, movement time, and ground reaction force) were assessed at pre-and post-training. The data were analyzed using a two-way mixed-design analysis of variance (ANOVA) (group × time). Both training programs improved the rate of force development in countermovement jump, the reaction time of punch, the peak ground reaction force of the rear leg during the jab punch, and the velocity of the jab punch. There were no group differences and interaction effects in all variables analyzed. It is concluded that 8 weeks of plyometric and rope jumping programs had a similar impact on improving lower extremity strength and punching performance. Both training programs may improve muscle strength and power, rate of force development, and reaction time. These improvements may contribute to lower extremity strength for driving a punch at the target with excellent performance.

## Highlights

• Plyometric training and jump rope are often used in boxing training for developing lower extremity ability.
• Both 8-week plyometric training and jump rope training can improve the peak RFD of lower extremity and jab punch performance (reaction time, peak GRF of the rear leg, and punch velocity) to a similar extent in junior amateur boxers.
• Punch forces during jab punch and cross punch were not significantly improved as reported in the experienced boxers. Differences in the maturity of boxing techniques between various levels of development may affect the effects of physical training.

## Introduction

Explosive power development is essential in many individual and team sports due to significantly enhancing performance (Loturco et al., 2018). In particular, in combat sports, generating explosive power in the upper body is required to deliver forceful punches to gain desired performance outcomes. Whilst the arm transmits the force at the end of the kinetic chain, the importance of the lower body in developing punch force should not be overlooked and should be taken into account when devising strength and conditioning training programs.

It has been demonstrated that increasing lower extremity power can result in greater punch force (Filimonov et al., 1985; Fritsche, 1987) with higher strength values observed inexperienced boxers (38.6%) compared with intermediate level (32.2%) and novice-level (16.5%) counterparts, respectively (Filimonov et al., 1985). This observation is supported by kinematic data, which has shown lower extremity force to differentiate the ability to drive a punch at high velocity (Dyson et al., 2007). For example, biomechanical analysis of a “straight of rear punch” has demonstrated punch power to increase (15%) due to the application of lower extremity force reinforcing the kinetic chain movement of the upper body (Tillin et al., 2010). Therefore, it is apparent that lower extremity explosive power development is a crucial consideration for strength and conditioning coaches who train boxers to develop punch power. Nevertheless, the different explosive lower extremity training programs effects on boxing punching performance remain unclear.

Plyometric training is a powerful method used in many sports, including boxing. It involves using quick and powerful jumps, hops, bounds, and skips utilizing the stretch-shortening cycle (Lloyd et al., 2012). Many studies suggest that plyometric training is practical training for lower extremities as it helps athletes enhance their performance in competition (de Villarreal et al., 2009; Goran & Pavle, 2010; Aboodarda et al., 2014; Chelly et al., 2014; George et al., 2015). However, its effects on the performance of boxers have not been well known.

Jumping rope is one of the most famous boxing exercises. It is a kind of stretch-shortening cycle movement in that the function of lower extremity muscles repeatedly extends and contracts in a short time (Miyaguchi et al., 2015). Jumping rope has been an effective training program for many sports like track and field, boxing, football, volleyball, basketball, and martial arts (Ozer et al., 2011). Performing jump rope during warm-up presents the advantage of both horizontal and vertical jumping tasks for track and field athletes (Makaruk, 2013). Many coaches believe that jump rope is a good way of training to build endurance and develop footwork and create strength and power to maintain performance in the boxing match.

Thus, the importance of studying intensely the kinetics and kinematics of the lower extremity movement has arisen. The boxer’s unique movement style, punches, and training are the key to success in amateur boxing. Specific training programs like plyometrics, ballistic, and weightlifting training are critical to enhancing the boxer’s muscle strength and power as it helps the lower extremity ability of the fighter to throw a punch at the target with great energy (Vidas et al., 2018). Therefore, improving lower extremity sports performance may contribute to punching performance in boxers. The researcher intends to investigate the usage of plyometric training protocols to enhance the performance of an amateur boxer. Therefore, this study compared the effects of 8-week plyometric training and jump rope training programs for improving lower extremity sports performance and punching performance in junior amateur boxers. We hypothesized that plyometric training programs would improve lower extremity sports performance and punching performance better than the jump rope training program. Moreover, an effective boxing program is needed to improve performance. Therefore, a faster, easier, and more effective training program is essential for the optimal design program.

## Materials and Methods

### Experimental Approach to the Problem

The testing protocol consists of biomechanics variables (leg stiffness, jump power, rate of force development) from a countermovement jump measurement. In addition, punching performance (punch velocity, punch force, reaction time, movement time, and ground reaction force) were also assessed using kinematic and kinetic analysis during a jab-cross punch movement. This investigation used a between-group repeated measures design in which all participants were evaluated before and after completing an 8-week lower extremity training program. Participants were randomly allocated into either an 8-week plyometric (PLY, *n* = 12) or jump rope (JR, *n* = 12) training group. Data were collected before and after each 8-week training program and included assessing lower extremity sports performance and punching performance.

### Participants

Twenty-four healthy male high school amateur boxers between the ages of 12 and 18 were recruited to participate in this study. The participants were amateur boxing their performed training at least 3 days per week. All Athletes have 3–5 years of experience in boxing training. No one of the participants has musculoskeletal or neurological conditions that prevent boxing training during participation. The participant characteristics are presented in Table 1. First, participants were informed of the potential risks and benefits of participating in the study. Then, the researcher obtained the consent form from the participants and their parents after fully explaining the experimental procedures. Finally, all procedures were performed according to the Declaration of Helsinki, and the University approved the study of Taipei Human Ethics Committee.

**TABLE 1:** Demographics of the participants.

	PLY	JR	*p*-value
	(*n* = 12)	(*n* = 12)	
	Mean ± SD	Mean ± SD	
Age (years)	15.5 ± 1.6	15.6 ± 1.6	0.912
Body Mass (kg)	57.8 ± 14.0	58.0 ± 12.9	0.737
Height (cm)	165.8 ± 8.1	167.6 ± 10.3	0.413
Body Mass Index (kg/m2)	20.8 ± 3.9	20.5 ± 3.5	0.802

### Training Protocol

The participants performed warm-up before the session, including 10-min dynamic exercises, multidirectional running, and active mobilization of all major muscle groups. The plyometric training program was based upon previous research (Lloyd et al., 2012) for 30 min per session and 3 days per week. It included lateral jumps, standing vertical jumps, horizontal jumps, ankle hopping, skipping, single leg hopping, and low-level hurdle drop jumps (20 cm). The intensity of plyometric training was progressed throughout the 8-week training period from 50 to 60 foot contacts in the first 4 weeks to 80–120-foot contacts from week five to eight. Therefore, the participants were asked to complete 6–8 repetitions of each exercise over 2–4 sets with a between-set rest interval of 2 min (Lloyd et al., 2012) (Table 2). In the JR group, jumping was simulated without the rope and using the arms to avoid confounding the recording of upper extremity punching performance parameters. The number of two-leg contacts and the number of sets were the same intensity as the PLY training program (Table 3). The training tempo in both exercise programs used a metronome rate of 120 beats per minute to ensure equal exercise intensity. Moreover, a researcher and an assistant boxing coach monitored all the training. The exercise compliance of each group was 100%.

**TABLE 2:** Plyometric program.

Exercise program	Week							
	1	2	3	4	5	6	7	8
1. Squat jump	2 × 6							
2. Countermovement jump	2 × 6	2 × 8						
3. Pogo jump	2 × 8	2 × 8	3 × 8	2 × 10	2 × 10	4 × 8	4 × 10	4 × 10
4. Standing long jump	2 × 8	4 × 4	2 × 3					
5. Lateral hops	2 × 8	4 × 8	4 × 8					
6. Hop scotch			3 × 4					
7. Bilateral power hop			4 × 3					
8. Ankle jump				3 × 5	3 × 5			
9. Power skipping				3 × 8	3 × 8	3 × 8		
10. Unilateral pogo hops				2 × 10	2 × 10	2 × 8	2 × 10	2 × 10
11. Max rebound hops				3 × 5	3 × 5	4 × 5	3 × 5	3 × 5
12. Drop jumps						2 × 5	4 × 4	4 × 4
13. Hurdle power hops							3 × 5	3 × 5
Total foot contacts	72	80	86	94	94	102	106	106

(sets x repetitions).

**TABLE 3:** Jumping rope program.

Exercise program	Week								
	1	2	3		4	5	6	7	8
Mimic jumping rope									
Total foot contacts	72	80	86	94	94	102	106	106

### Biomechanical Analysis of Punching and Lower Extremity Sports Performance

**FIGURE 1:**
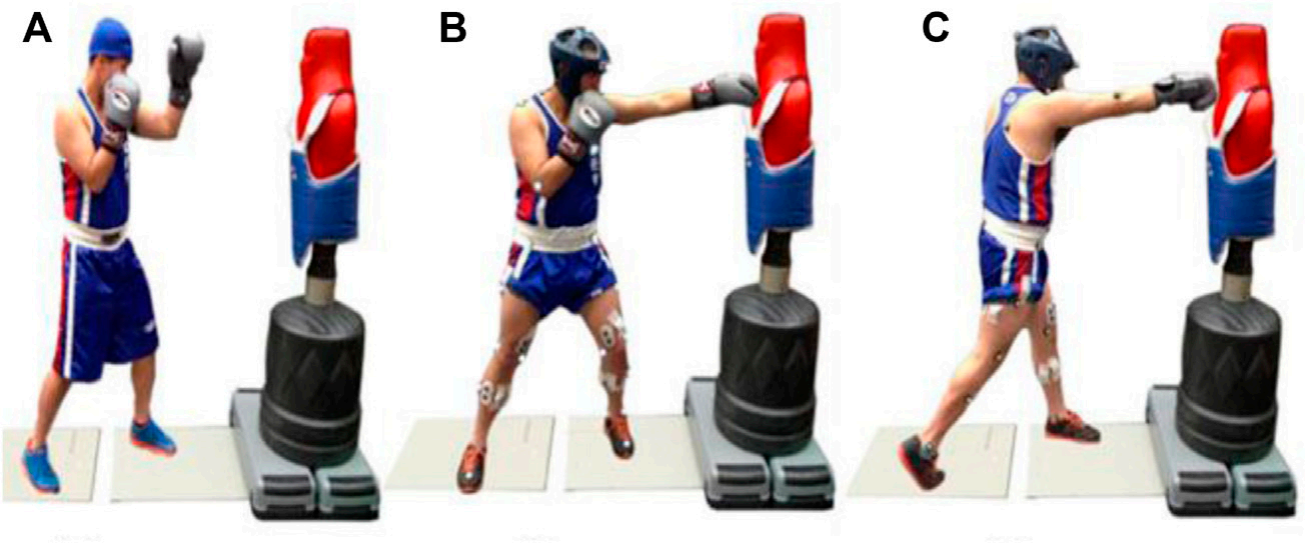
Punching assessment, **(A)** start to landing; **(B)** landing to jab; **(C)** jab to cross.

Biomechanical data were collected before and after the completion of the training programs. Participants were asked to avoid intense physical activity within the 24 h before the test measurements. Before testing, participants were required to perform a 10 min standardized dynamic warm-up. The punching performance assessment was undertaken on two force platforms (AMTI, Inc., Newton, MA) using a sampling frequency of 1,000 Hz. All data were low pass filtered using a 4th order Butterworth filter with a cut-off frequency of 40 Hz. For synchronization of the force and motion analysis data, the program marked with a light trigger is stuck on the dummy, visible to the camera’s field of view. Participants were asked to stand with the lead leg on the first force plate and the rear leg on the second force plate. The participants were not informed of the stimulus’s appearance on the punch target; however, they received a warning (“ready”) to be prepared when the program started. Participants were asked to punch as fast as possible when the light stimulus appeared, requiring the lead leg to step forward onto the force platform to jab punch, followed by a rapid cross punch to the target. The punching performance assessment adjusted the displacement to the target according to the participant’s arm distance to avoid leaning forward, increasing the force on the target. Participants performed 5 jab-cross forces with 1-minute rest between trials (Figure 1). Twenty-nine reflective markers fixed on body landmarks (Helen Hayes model) were calculated punch velocity (PV), 10 high-speed infrared cameras recorded with a frequency of 250 Hz (Motion Analysis System, Santa Rosa, California, United States) During the trials, a tri-axial accelerometer (Model SS34L, BIOPAC System, 42 Aero Camino Goleta, CA) with a sampling frequency of 3,000 Hz was attached to the target dummy with neotape covering the sensor.

The axis orientation was fixed to the target dummy, with the x-axis parallel to the forward of the horizontal plane and used to identify punch force (PF) and the time of movement (MT). MT was recorded as the duration between the lead leg leaving the force platform and the signal’s appearance in the target dummy’s accelerometer. The RMS acceleration is the square root of the area under the ASD curve in the frequency domain, and the data were converted to velocity data using numerical integration in the trapezoidal rule (Dennis & David, 2015). Reaction time (RT) was recorded by the interval of time to illustrate the stimulus and initiation response of the lead leg. The ground exerts the ground reaction force (GRF) on a body in contact. GRF was collected from the lower extremities of the participant using the two embedded force platforms. The sampling rate was set at 1,000 Hz, and the force plate reaction time (FPRT) data was extracted by identifying the initial ground contact and take-off time from the lead leg. The average of the five jab-cross punch trials on the force plate was used in the subsequent analysis.

For the lower extremity sports performance assessment, participants were directed to perform the countermovement jump (CMJ), emphasizing a quick jump, as a sharp transition between eccentric to concentric movement is essential to the stretch-shortening cycle (Morin et al., 2005; Nicol et al., 2006; Anthony, 2010). Participants performed 10 min dynamic warm-up, including light jogging and dynamic stretching. Then, they conducted five practice CMJ trials of increasing intensity after 5 min following the warm-up. Finally, they completed 5 CMJ trials with 90 s rest between shots for data collection. Tests were collected using 10 high-speed cameras (Motion Analysis System, Santa Rosa, California, United States) sampled at 250 Hz, and an AMTI force platform (AMTI, Inc., Newton, MA) sampled at 1,000 Hz. The participants were conducted to stand upright on the force platform with weight equally distributed over both feet. The participants were performed to stand with arms akimbo and be in the same position throughout the trial. When all was ready and the visual stimulus lit up from the initial starting position, participants performed squat downward movement immediately followed by a quick and forceful jump to take off from the ground. The rate of force development (RFD) is a measure of explosive strength, for the CMJ was calculated by the force difference between the rising point and the peak point of the ground reaction force (GRF) divided by the time difference of these two-time points (ΔForce/Δtime) (Ebben et al., 2008). Jump power (JP) was the peak value of instantaneous power (the product of instantaneous GRF and velocity in a vertical direction) during the entire period of each jump. Vertical leg stiffness (VLS) was calculated using the spring-mass model (Hobara et al., 2012; Miyaguchi et al., 2014b). It was calculated by dividing the peak vertical GRF to the center of mass (COM) displacement between the highest and the lowest point during the CMJ trial. All markers calculated COM from the body (Helen Hayes model) during jumping with Orthotrak program (ORTHOTRAK 6.2.4; Single Trial Processing Module, Motion Analysis System; Clinical Gait Analysis Software) (Morin et al., 2005; Miyaguchi et al., 2014b; Miyaguchi et al., 2015).

### Statistical Analysis

The means and standard deviations or 95% Confidence Intervals (95%CI) for each outcome measure are shown. The normality assumption was confirmed using a Shapiro-Wilk test to ensure the normal distribution of the quantitative data (*p* > 0.05). Comparison of the baseline data between groups using an independent t-test. For training-induced changes, the student’s t-test was used to identify differences at baseline, while a repeated measure two-way ANOVA was applied to assess the occurrence of an actual training effect, *p* < 0.05 for the interaction (group × time) on the punching performance and lower extremity force. The sample size was determined with G*power (version 3.1.9.4) software. The calculations were based on detecting an effect size of 0.9 based on changes in jab velocity in a prior study (Dennis & David, 2015), an α level of 5%, and the desired power of 90%. These assumptions generated the desired sample size of at least 12 subjects per group. All statistical analyses were performed with SPSS statistical software version 26.0 (IBM Corp., United States). The statistical analysis was conducted at a 95% confidence level. A *p*-value of < 0.05 was considered statistically significant for all studies.

## Results

### Punching Performance

Jab-cross punch biomechanical characteristics are shown in Table 4. The results revealed a statistically significant main effect of time for jab punch velocity, F (1,22) = 4.806, *p* = 0.039. On the other hand, there was no significant main effect of group, F (1,22) = 0.044, *p* = 0.836. Moreover, there was no significant interaction between types of training and time effect F (1,22) = 1.160, *p* = 0.239. On the other hand, there was a significant main effect of time for reaction time, F (1,22) = 4.202, *p* = 0.047. Then, there was no significant main effect of group, F (1,22) = 0.271, *p* = 0.608. Additionally, there was no significant interaction between types of training and time effect, F (1,22) = 0.140, *p* = 0.712.

**TABLE 4:** Biomechanical characteristics of jab-cross punch before and after PLY or J.R. training.

	Test	PLY	JR	F (df)	Time effect	Group effect	Group × Time Interaction
		(*n* = 12)	(*n* = 12)				
		Mean ± SD with 95% CI	Mean ± SD with 95% CI	*p*-value			
Jab punch velocity	Pre	4.2 ± 0.5 (3.8–4.5)	4.3 ± 0.6 (3.8–4.7)	F (1,22)	4.806	0.044	1.160
(m/s)	Post	4.6 ± 0.4 (4.3–4.9)	4.5 ± 0.7 (3.9–4.9)	*p*-value	0.039*	0.836	0.293
Cross punch velocity	Pre	6.8 ± 0.9 (6.2–7.4)	6.4 ± 1.0 (5.7–7.1)	F (1,22)	0.100	1.197	0.150
(m/s)	Post	6.9 ± 0.7 (6.4–7.4)	6.4 ± 1.1 (5.6–7.1)	*p*-value	0.755	0.286	0.702
Jab punch force	Pre	6.7 ± 0.6 (6.3–7.1)	7.2 ± 1.5 (6.2–8.1)	F (1,22)	0.934	0.503	0.457
(g)	Post	7.3 ± 0.9 (6.7–7.8)	7.3 ± 1.4 (6.4–8.2)	*p*-value	0.342	0.486	0.506
Cross punch force	Pre	10.1 ± 2.7 (8.3–11.8)	9.1 ± 1.7 (7.9–10.2)	F (1,22)	0.486	2.564	0.171
(g)	Post	10.5 ± 1.7 (9.3–11.6)	9.2 ± 1.0 (8.5–9.8)	*p*-value	0.493	0.124	0.684
Reaction time	Pre	0.24 ± 0.1 (0.18–0.30)	0.25 ± 0.1 (0.17–0.33)	F (1,22)	4.202	0.271	0.140
(s)	Post	0.20 ± 0.03 (0.17–0.22)	0.22 ± 0.1 (0.16–0.28)	*p*-value	0.047*	0.608	0.712
Movement time	Pre	0.64 ± 0.1 (0.58–0.69)	0.66 ± 0.1 (0.59–0.73)	F (1,22)	0.165	2.076	0.390
(s)	Post	0.61 ± 0.1 (0.55–0.67)	0.67 ± 0.1 (0.61–0.72)	*p*-value	0.689	0.164	0.539

*p < 0.05.

Values are given as mean ± SD. A 2-way analysis of variance with repeated measure (group x time) was used to assess training-related effects’ statistical significance.

PLY, plyometric; JR, jump rope, m/s = meter per second, s = second and g = G-forces.

### Peak Ground Reaction Force

Significant time effects for both training methods were observed in the peak GRF of the rear leg during the landing to jab phase, F (1,22) = 6.903, *p* = 0.015. No significant main effect of group, F (1,22) = 2.839, *p* = 0.106. Furthermore, there was no significant interaction between type of training and time effect, F (1,22) = 3.110, *p* = 0.092 (Table 5).

**TABLE 5:** Lead leg and rear leg ground reaction force during jab-cross punch before and after PLY or J.R. training.

	Test	PLY	RJ	F (df)	Time effect	Group Effect	Group x Time Interaction
		(*n* = 12)	(*n* = 12)				
		Mean ± SD with 95% CI	Mean ± SD with 95% CI	*p*-value			
1.Start to landing phase							
Lead leg PGRF (BW)	Pre	0.56 ± 0.08 (0.5–0.6)	0.55 ± 0.07 (0.49–0.59)	F (1,22)	0.141	0.079	0.002
	Post	0.55 ± 0.09 (0.48–0.6)	0.54 ± 0.07 (0.49–0.58)	*p*-value	0.739	0.781	0.967
Rear leg PGRF (BW)	Pre	1.33 ± 0.30 (1.11–1.49)	1.28 ± 0.17 (1.14–1.37)	F (1,22)	0.000	0.135	0.110
	Post	1.31 ± 0.28 (1.11–1.46)	1.30 ± 0.21 (1.14–1.42)	*p*-value	0.983	0.716	0.743
2.Landing to jab phase							
Lead leg PGRF (BW)	Pre	0.48 ± 0.22 (0.33–0.61)	0.50 ± 0.16 (0.38–0.58)	F (1,22)	0.042	0.011	0.064
	Post	0.50 ± 0.19 (0.36–0.61)	0.49 ± 0.13 (0.40–0.57)	*p*-value	0.840	0.917	0.803
Rear leg PGRF (BW)	Pre	0.77 ± 0.18 (0.64–0.87)	0.77 ± 0.17 (0.64–0.86)	F (1,22)	6.903	2.839	3.110
	Post	1.03 ± 0.22 (0.87–1.15)	0.82 ± 0.25 (0.64–0.96)	*p*-value	0.015*	0.106	0.092
3.Jab to cross phase							
Lead leg PGRF (BW)	Pre	1.07 ± 0.23 (0.91–1.20)	1.09 ± 0.18 (0.95–1.18)	F (1,22)	0.387	0.007	0.091
	Post	1.10 ± 0.20 (0.94–1.20)	1.10 ± 0.16 (0.97–1.17)	*p*-value	0.540	0.936	0.765
Rear leg PGRF (BW)	Pre	0.66 ± 0.24 (0.49–0.80)	0.66 ± 0.27 (0.47–0.81)	F (1,22)	0.049	0.001	0.002
	Post	0.67 ± 0.23 (0.51–0.80)	0.66 ± 0.23 (0.50–0.80)	*p*-value	0.826	0.979	0.961

*p < 0.05.

Values are given as mean ± S.D. A 2-way analysis of variance with repeated measure (group x time) was used to assess training-related effects’ statistical significance.

BW, body weight; PLY, plyometric; JR, jump rope and PGRF, peak ground reaction force.

### Lower Extremity Strength

There was a significant main effect of time for the peak rate of force development (PRFD) in the Countermovement jump, F (1,22) = 4.640, *p* = 0.042. In contrast, there was no significant main effect of group, F (1,22) = 0.189, *p* = 0.668 and interaction effect, F (1,22) = 2.494, *p* = 0.129 (Table 6).

**TABLE 6:** Biomechanical characteristics of countermovement jump before and after PLY or J.R. training.

	Test	PLY	JR	F (df)	Time effect	Group effect	Group × Time Interaction
		(*n* = 12)	(*n* = 12)				
		Mean ± SD with 95% CI	Mean ± SD with 95% CI	*p*-value			
Vertical leg stiffness (kN/m)	Pre	71.3 ± 13.4	73.3 ± 21.7	F (1,22)	0.141	0.193	0.044
		(62.7–79.8)	(59.4–87.1)				
	Post	69.7 ± 15.6	72.8 ± 10.5	*p*-value	0.711	0.665	0.837
		(59.7–79.6)	(66.1–79.5)				
Jump power (W)	Pre	5,357.6 ± 678.0	5,102.0 ± 202.0	F (1,22)	1.272	0.158	0.755
		(3,234.3–7,480.8)	(3,504.9–6,699.1)				
	Post	5,886.0 ± 895.0	5,170.5 ± 205.1	*p*-value	0.272	0.695	0.394
		(3,572.6–8,199.3)	(3,523.1–6,817.9)				
PRFD (N/s)	Pre	7,737.10 ± 1,538.2	7,854.6 ± 1,361.3	F (1,22)	4.646	0.189	2.494
		(6,697.2–8.776.8)	(6,989.6–8,719.6)				
	Post	8,607.0 ± 1,719.0	7,988.6 ± 1,329.8	*p*-value	0.042*	0.668	0.129
		(7,514.8–9,699.2)	(7,143.6–8,833.5)				
ARFD (N/s)	Pre	4,652.1 ± 1,239.0	4,419.9 ± 1,379.3	F (1,22)	0.071	0.461	0.189
		(3,864.8–5,439.3)	(3,406.2–5,433.5)				
	Post	4,686.9 ± 637.5	4,274.9 ± 1,395.0	*p*-value	0.792	0.504	0.668
		(4,281.8–5,092.0)	(3,388.3–5,161.1)				

*p < 0.05.

Values are given as mean ± S.D. A 2-way analysis of variance with repeated measure (group x time) was used to assess training-related effects’ statistical significance.

PLY, plyometric; JR, jump rope; PRFD, peak rate of force development and ARFD, average rate of force development, kN/m = kilonewton per meter, W = watts and N/s = newton per second.

## Discussion

This study aimed to investigate the effects of two different exercise programs on lower extremity sports performance and punching performance in high school amateur boxers. The results from the investigation show no significant difference in lower extremity sports performance and punching performance between the PLY and JR training programs. In addition, we observed no conferred advantage of selecting either training program to enhance any measured biomechanical or performance parameter.

The study compared JR and PLY because both training exercises’ characteristics are similar. The movement of the feet makes contact with the ground and the lower extremity during the stretch-shortening cycle. During SSC, muscles, especially the ankle, knee, and hip joints, are activated in a specific mode under eccentric contraction, producing high muscle forces and elastic energy storage greater than during isometric or concentric contraction (Gruber et al., 2019). In addition, plyometrics are a natural part of most movement, as evidenced by jumping, hopping, and skipping (George et al., 2015).

In both groups, the velocity of the jab punch was improved after completing the 8-week training program. It is possible that the effects of the plyometric and jump rope training reduced the time to complete stretch-shortening cycles (Anthony, 2010; Miyaguchi et al., 2014b), enhancing the eccentric to concentric phase of contraction in the lower extremities. This may have decreased reaction time and increased peak GRF of the rear leg during the jab punch (Irineu et al., 2010). Consequently, the observed increase in jab punch velocity may reflect the gain in peak power output from the lower extremities (Chelly et al., 2014). Alternatively, or in combination, the improvement in punch velocity may have been related to an increase in fast-twitch fiber cross-sectional area, improved neural activation, changes in intrinsic muscular properties, an increase in myosin-ATP activity, better synchronization or higher firing frequency of motor units (Gorostiaga et al., 2006). However, we were not able to examine these changes. Nevertheless, the ability of the lower extremities to improve punch velocity may also have been associated with changing kinetic and kinematic characteristics of the boxing technique.

There has been limited research undertaken to investigate the effects of JR and plyometric training to develop the RFD of boxers. In this study, a marked increase in peak RFD was observed in both training groups after 8 weeks of training. Our findings agree with previous work, demonstrating that an acute period of plyometric training may improve RFD compared to traditional weight training (Kramer et al., 1993). However, in the present study, the increase in RFD was likely related to the enhanced coordination and the ability to rapidly increase muscle loading due to plyometric activity (Kramer et al., 1993). In contrast, no significant improvement in peak RFD has been reported after weightlifting training (Guy et al., 2008), combining power training with heavy loads (Lamas et al., 2012), hypertrophy training (Loturco et al., 2014), maximal voluntary contractions (de Oliveira et al., 2013) or after a periodized strength period, which includes hypertrophy and power training phases (Hartmann et al., 2009).

The transmission of lower extremity increased force through the upper extremity allows for more incredible velocity during punching (Marshall et al., 2011). Experienced boxers may increase this forward movement (rapidly lift the lead leg) by simultaneously pushing off with more rear leg extension (Anthony, 2011). Furthermore, decreasing the duration of the amortization phase exploits the stored elastic energy and the stretch reflex, allowing a greater power during the concentric phase of the movement (de Villarreal et al., 2009). Therefore, this study’s more excellent rear leg GRF may permit more incredible punch velocity.

Previously, it has been documented that JR effectively improves the 20 m sprint ability (Miyaguchi et al., 2015). However, like sprinting, the leg drive during punching requires GRF to be vertically and horizontally. Consequently, depending on the primary direction of the GRF during punching, it may be more appropriate to emphasize longitudinal movements, such as with jumps (Lenetsky et al., 2013). This may be speculated to be due to an improved motor ability and body balance enhancing perceptual and physical factors (Fleishman et al., 1984).

The study’s limitations were relative human participation and research methodology. First, we intended to determine our investigation of high-performance junior amateur boxers, which inevitably leads to a low number of participants and is typical in studies of specific professional populations, especially junior amateur boxing athletes. It is difficult to determine the volume of sports activity in the school because of the training load used to conduct an equivalent intensity of exercise program. Then, a limitation of the present study was that the punch force generated by the rear leg might have resulted in a different impact force while fighting an opponent because the different trunk angles and distances are likely to be employed when throwing a punch at an opponent (Hickey, 1980). In addition, since we used junior high school boxers as participants, the ceiling capacity to develop lower extremity power is likely lower than more experienced boxers. Therefore, it remains unclear whether similar improvements in biomechanical parameters would be noted after completing the same training programs for professional boxers. Finally, while we attempted to control the volume and intensity of both training programs, another non-programmed activity was difficult to control. Future studies may use cross-over study methods to investigate amateur boxers’ lower extremity strength and punching performance. However, it is expected that these points will help future researchers avoid facing the same shortcomings.

## Conclusion

Our study demonstrates that performing an 8-week program of either PLY or JR training may be able to achieve more improvements in both punching performance and lower extremity strength to a similar extent. Therefore, additional training programs like plyometric or jumping rope training may improve the muscle strength and power, rate of force development, and reaction time as it helps in the lower extremity strength to drive a punch at the target with excellent performance.

### Practical Application

There is no control group in this study. The interpretation of the findings should be careful. However, research showed that increasing lower extremity power can result in greater punch force observed inexperienced boxers. Plyometric training and jump rope are often used in boxing training for developing lower extremity ability. However, their effects on the boxing performance were rarely reported, mainly applied to the junior amateur boxers. This study showed that both 8-week plyometric and jump rope training could improve the peak RFD of lower extremity and jab punch performance (reaction time, peak GRF of the rear leg, and punch velocity) to a similar extent in junior amateur boxers. However, punch forces during jab punch and cross punch were not significantly improved as reported in the experienced fighters in previous studies. Differences in the maturity of boxing techniques between various levels of development may affect the effects of physical training. The efficiency of the kinetic chain while transferring forces from the lower body through to the fist should be considered.

## Data Availability

The raw data supporting the conclusions of this article will be made available by the authors, without undue reservation.

## References

[B1] Aboodarda S. J. Byrne J. M. Samson M. Wilson B. D. Mokhtar A. H. Behm D. G. (2014). Does Performing Drop Jumps with Additional Eccentric Loading Improve Jump Performance? J. Strength Cond. Res. 28 (8), 2314–2323. 10.1519/JSC.0000000000000498 24796986

[B3] Bruzas V. Kamandulis S. Venckunas T. Snieckus A. Mockus P. (2018). Effects of Plyometric Exercise Training with External Weights on Punching Ability of Experienced Amateur Boxers. J. Sports Med. Phys. Fit. 58 (3), 221–226. 10.23736/S0022-4707.16.06674-3 27623756

[B4] Chelly M. S. Hermassi S. Aouadi R. Shephard R. J. (2014). Effects of 8-Week In-Season Plyometric Training on Upper and Lower Limb Performance of Elite Adolescent Handball Players. J. Strength Cond. Res. 28 (5), 1401–1410. 10.1519/JSC.0000000000000279 24149768

[B6] de Oliveira F. B. D. Rizatto G. F. Denadai B. S. (2013). Are Early and Late Rate of Force Development Differently Influenced by Fast-Velocity Resistance Training? Clin. Physiol. Funct. Imaging 33 (4), 282–287. 10.1111/cpf.12025 23692617

[B7] de Villarreal E. S.-S. Kellis E. Kraemer W. J. Izquierdo M. (2009). Determining Variables of Plyometric Training for Improving Vertical Jump Height Performance: A Meta-Analysis. J. Strength Cond. Res. 23 (2), 495–506. 10.1519/JSC.0b013e318196b7c6 19197203

[B9] Dyson R. Smith M. Martin C. Fenn L. (2007). “Muscle Recruitment during Rear Hand Punches Delivered at Maximal Force and Speed by Amateur Boxers,” in 25 International Symposium on Biomechanics in Sports, Ouro Preto, Brazil, 2007, August 23-27.

[B10] Ebben W. P. Flanagan E. P. Jensen R. L. (2008). Jaw Clenching Results in Concurrent Activation Potentiation during the Countermovement Jump. J. Strength Cond. Res. 22 (6), 1850–1854. 10.1519/JSC.0b013e3181875117 18978622

[B11] Filimonov V. I. Koptsev K. N. Husyanov Z. M. Nazarov S. S. (1985). Boxing: Means of Increasing Strength of the Punch. Natl. Strength Cond. Assoc. J 7 (6), 65–66. 10.1519/0744-0049(1985)007<0065:moisot>2.3.co;2

[B12] Fleishman E. A. Quaintance M. K. Broedling L. A. (1984). Taxonomies of Human Performance: The Description of Human Tasks. Academic Press.

[B13] Fritsche P. (1987). Ein dynamographisches Informationssystem zur Messung der Schlagkraft beim Boxen. Leistungssport 2, 151–156.

[B14] George D. Bryan L. R. Robert M. (2015). Current Concepts of Plyometric Exercise. Int. J. Sports Phys. Ther. 10 (6), 760–786. 26618058 PMC4637913

[B15] Gorostiaga E. M. Granados C. Ibañez J. González-badillo J. J. Izquierdo M. (2006). Effects of an Entire Season on Physical Fitness Changes in Elite Male Handball Players. Med. Sci. Sports Exerc. 38 (2), 357–366. 10.1249/01.mss.0000184586.74398.03 16531907

[B16] Gruber M. Kramer A. Mulder E. Rittweger J. (2019). The Importance of Impact Loading and the Stretch Shortening Cycle for Spaceflight Countermeasures. Front. Physiol. 10. 10.3389/fphys.2019.00311 PMC6438856 30967797

[B17] Guy G. H. Burgess S. J. Michael H. S. (2008). Training: Theoretical and Practical Applications for the Strength and Conditioning Professional. U.K. Strength Cond. Assoc. 12, 12–16.

[B18] Hartmann H. Bob A. Wirth K. Schmidtbleicher D. (2009). Effects of Different Periodization Models on Rate of Force Development and Power Ability of the Upper Extremity. J. Strength Cond. Res. 23 (7), 1921–1932. 10.1519/JSC.0b013e3181b73c69 19855316

[B19] Hickey K. (1980). Boxing-The Amateur Boxing Association Coaching Manual. Kaye and Ward.

[B21] Hobara H. Kato E. Kobayashi Y. Ogata T. (2012). Sex Differences in Relationship between Passive Ankle Stiffness and Leg Stiffness during Hopping. J. Biomechanics 45, 2750–2754. 10.1016/j.jbiomech.2012.09.008 23051683

[B22] Kimm D. Thiel D. V. (2015). Hand Speed Measurements in Boxing. Procedia Eng. 112, 502–506. 10.1016/j.proeng.2015.07.232

[B23] Kramer J. Morrow A. Leger A. (1993). Changes in Rowing Ergometer, Weight Lifting, Vertical Jump and Isokinetic Performance in Response to Standard and Standard Plus Plyometric Training Programs. Int. J. Sports Med. 14, 449–454. 10.1055/s-2007-1021209 8300271

[B24] Lamas L. Ugrinowitsch C. Rodacki A. Pereira G. Mattos E. C. T. Kohn A. F. (2012). Effects of Strength and Power Training on Neuromuscular Adaptations and Jumping Movement Pattern and Performance. J. Strength Cond. Res. 26, 3335–3344. 10.1519/JSC.0b013e318248ad16 22222321

[B25] Lenetsky S. Harris N. Brughelli M. (2013). Assessment and Contributors of Punching Forces in Combat Sports Athletes. Journal 35 (2), 1–7. 10.1519/SSC.0b013e31828b6c12

[B26] Lloyd R. S. Oliver J. L. Hughes M. G. Williams C. A. (2012). The Effects of 4-Weeks of Plyometric Training on Reactive Strength Index and Leg Stiffness in Male Youths. J. Strength Cond. Res. 26, 2812–2819. 10.1519/JSC.0b013e318242d2ec 22130392

[B27] Loturco I. Artioli G. G. Kobal R. Gil S. Franchini E. (2014). Predicting Punching Acceleration from Selected Strength and Power Variables in Elite Karate Athletes. J. Strength Cond. Res. 28, 1826–1832. 10.1519/JSC.0000000000000329 24276310

[B28] Loturco I. Bishop C. Ramirez-Campillo R. Romano F. Alves M. Pereira L. (2018). Optimum Power Loads for Elite Boxers: Case Study with the Brazilian National Olympic Team. Sports 6 (3), 95. 10.3390/sports6030095 PMC6162793 30217089

[B29] Loturco I. Pereira L. A. Kobal R. Maldonado T. Piazzi A. F. Bottino A. (2010). Improving Sprint Performance in Soccer: Effectiveness of Jump Squat and Olympic Push Press Exercises. PLoS One 11 (4), e0153958–12. 10.1371/journal.pone.0153958 PMC4839661 27100085

[B31] Makaruk H. (2013). Acute Effects of Rope Jumping Warm-Up on Power and Jumping Ability in Track and Field Athletes. Pol. J. Sport Tour. 20, 200–204. 10.2478/pjst-2013-0018

[B32] Markovic G. Mikulic P. (2010). Neuro-Musculoskeletal and Performance Adaptations to Lower-Extremity Plyometric Training. Sports Med. 40 (10), 859–895. 10.2165/11318370-000000000-00000 20836583

[B33] Marshall P. W. M. McEwen M. Robbins D. W. (2011). Strength and Neuromuscular Adaptation Following One, Four, and Eight Sets of High Intensity Resistance Exercise in Trained Males. Eur. J. Appl. Physiol. 111 (12), 3007–3016. 10.1007/s00421-011-1944-x 21451937

[B34] Miyaguchi K. Demura S. Omoya M. (2015). Relationship between Jump Rope Double Unders and Sprint Performance in Elementary Schoolchildren. J. Strength Cond. Res. 29 (11), 3229–3233. 10.1519/JSC.0000000000000543 24852257

[B35] Miyaguchi K. Sugiura H. Demura S. (2014b). Possibility of Stretch-Shortening Cycle Movement Training Using a Jump Rope. J. Strength Cond. Res. 28, 700–705. 10.1519/JSC.0b013e3182a0c9a5 23860284

[B36] Morin J.-B. Dalleau G. Kyröläinen H. Jeannin T. Belli A. (2005). A Simple Method for Measuring Stiffness during Running. J. Appl. Biomechanics 21 (2), 167–180. 10.1123/jab.21.2.167 16082017

[B37] Nicol C. Avela J. Komi P. V. (2006). The Stretch-Shortening Cycle. Sports Med. 36 (11), 977–999. 10.2165/00007256-200636110-00004 17052133

[B38] Ozer D. Duzgun I. Baltaci G. Karacan S. Colakoglu F. (2011). The Effects of Rope or Weighted Rope Jump Training on Strength, Coordination and Proprioception in Adolescent Female Volleyball Players. J. Sports Med. Phys. Fit. 51, 211–219. 21681154

[B41] Tillin N. A. Jimenez-Reyes P. Pain M. T. G. Folland J. P. (2010). Neuromuscular Performance of Explosive Power Athletes versus Untrained Individuals. Med. Sci. Sports Exerc. 42 (4), 781–790. 10.1249/MSS.0b013e3181be9c7e 19952835

[B42] Turner A. Baker E. Miller S. (2011). Increasing the Impact Force of the Rear Hand Punch. J. Strength Cond. Res. 33 (6), 2–9. 10.1519/SSC.0b013e318232fdcb

[B43] Turner A. N. Jeffreys I. (2010). The Stretch-Shortening Cycle: Proposed Mechanisms and Methods for Enhancement. J. Strength Cond. Res. 32 (6), 87–99. 10.1519/SSC.0b013e3181e928f9

